# Establishing Effectiveness of a Community-based, Physical Activity Program for Fathers and Daughters: A Randomized Controlled Trial

**DOI:** 10.1093/abm/kaab056

**Published:** 2021-07-07

**Authors:** Philip J Morgan, Anna T Rayward, Myles D Young, Emma R Pollock, Narelle Eather, Alyce T Barnes, Stevie-Lee Kennedy, Kristen L Saunders, Ryan J Drew, David R Lubans

**Affiliations:** Priority Research Centre for Physical Activity and Nutrition, School of Education, Faculty of Education and Arts, University of Newcastle, New South Wales, Australia; School of Education, College of Human and Social Futures, University of Newcastle, New South Wales, Australia; Priority Research Centre for Physical Activity and Nutrition, School of Education, Faculty of Education and Arts, University of Newcastle, New South Wales, Australia; School of Education, College of Human and Social Futures, University of Newcastle, New South Wales, Australia; Priority Research Centre for Physical Activity and Nutrition, School of Education, Faculty of Education and Arts, University of Newcastle, New South Wales, Australia; School of Psychology, College of Engineering, Science and Environment, University of Newcastle, New South Wales, Australia; Priority Research Centre for Physical Activity and Nutrition, School of Education, Faculty of Education and Arts, University of Newcastle, New South Wales, Australia; School of Education, College of Human and Social Futures, University of Newcastle, New South Wales, Australia; Priority Research Centre for Physical Activity and Nutrition, School of Education, Faculty of Education and Arts, University of Newcastle, New South Wales, Australia; School of Education, College of Human and Social Futures, University of Newcastle, New South Wales, Australia; Priority Research Centre for Physical Activity and Nutrition, School of Education, Faculty of Education and Arts, University of Newcastle, New South Wales, Australia; School of Education, College of Human and Social Futures, University of Newcastle, New South Wales, Australia; Priority Research Centre for Physical Activity and Nutrition, School of Education, Faculty of Education and Arts, University of Newcastle, New South Wales, Australia; School of Education, College of Human and Social Futures, University of Newcastle, New South Wales, Australia; Priority Research Centre for Physical Activity and Nutrition, School of Education, Faculty of Education and Arts, University of Newcastle, New South Wales, Australia; School of Education, College of Human and Social Futures, University of Newcastle, New South Wales, Australia; Priority Research Centre for Physical Activity and Nutrition, School of Education, Faculty of Education and Arts, University of Newcastle, New South Wales, Australia; School of Education, College of Human and Social Futures, University of Newcastle, New South Wales, Australia; Priority Research Centre for Physical Activity and Nutrition, School of Education, Faculty of Education and Arts, University of Newcastle, New South Wales, Australia; School of Education, College of Human and Social Futures, University of Newcastle, New South Wales, Australia

**Keywords:** Exercise, Girls, Men, Fundamental movement skills, Parenting, Community trial

## Abstract

**Background:**

The ‘Dads And Daughters Exercising and Empowered’ (DADEE) program significantly improved physical activity levels of fathers and their daughters in an efficacy trial. However, the effectiveness of interventions when delivered in real-world settings needs to be established.

**Purpose:**

To evaluate the effectiveness of the DADEE intervention when delivered in community settings by trained facilitators.

**Methods:**

We conducted a two-arm RCT, (baseline and 3-months post-intervention assessments), in Newcastle, Australia. In 2016, 155 fathers (27–60 years) and 189 primary-school-aged daughters (4–12 years) (*n* = 344) were randomly allocated to the intervention (78 fathers, 95 daughters) or waitlist-control (77 fathers, 94 daughters) groups. Trained facilitators delivered the 9-week DADEE program (weekly sessions plus home-based tasks). Primary outcomes were fathers’ and daughters’ physical activity (steps/day). Secondary outcomes included screen-time, weight status, daughters’ fundamental movement skill (FMS) proficiency, perceived sports competence, and fathers’ parenting practices. Effects were assessed using linear mixed models.

**Results:**

Primary outcome follow-up data were collected from 88% of fathers and 89% of daughters. Significant group-by-time differences in mean daily steps were found for fathers’ (adjusted difference = +1,638; 95% CI: 833, 2,443, *d* = 0.7) and daughters’ (adjusted difference = +1,023 steps/day; 95% CI: 259, 1,787*; d* = 0.4) physical activity. Significant effects were observed for daughters’ screen-time, FMS, and some parenting practices. No significant effects were identified for weight status, or fathers’screen-time or self-reported MVPA. Program attendance, satisfaction and fidelity were very high.

**Conclusion:**

This study established the effectiveness of the DADEE intervention when delivered in community settings by trained facilitators. Importantly, the findings were comparable to those of the efficacy RCT delivered by the research team. To maximize public health benefits, a larger-scale dissemination of the program appears warranted.

Trial Registration Australian New Zealand Clinical Trial Registry: ACTRN12616001270404 Human Research Ethics Committee: H-2014-0330

## Teaser

A father-daughter physical activity intervention improved physical activity levels of both fathers and daughters when delivered by trained facilitators in community settings.

Physical activity is essential for children, providing benefits to physical, mental, social, and cognitive health [1–3]. However, approximately 80% of youth worldwide fail to meet the minimum physical activity guidelines [4, 5] of 60 min per day of moderate-to-vigorous-intensity physical activity [6]. Girls are less physically active than boys [2], with the disparity widening with age [7]. There are many socio-ecological factors, including societal norms and prejudices relating to gender stereotypes [8, 9], which negatively influence girls’ physical activity opportunities, attitudes, and behaviors. Consequently, girls have lower fitness levels and fundamental movement skill (FMS) proficiency than boys [9]. FMS proficiency is considered to provide a foundation for an active lifestyle [10], yet <5% of primary school-aged girls have mastered key skills including striking, dribbling, overhand throwing, and kicking [11]. Therefore, primary-school aged girls are at risk of the associated negative implications for health and participation in physical activity throughout life [10]. However, previous interventions to improve girls’ physical activity levels have generally had limited success [12, 13].

Parents have a significant influence on the activity levels of their children through their role modeling, parental practices and their role in shaping the home physical activity environment [14, 15]. Although girls’ participation in physical activity is predicted by activity levels of both mothers and fathers [16], fathers tend to spend more time encouraging and co-participating in physical activity with sons rather than daughters [15]. Furthermore, fathers are less likely to meet physical activity recommendations than men without children and so also need opportunities to increase their physical activity levels [17, 18]. However, fathers are greatly under-represented as agents of change in family-based pediatric obesity prevention and treatment interventions, accounting for only 6% of participating parents [19]. Therefore, there is a strong need to meaningfully engage fathers in family-based physical activity interventions [18].

To address the absence of fathers in parenting interventions and the call for innovative approaches to improve girls’ physical activity levels [19], we conducted the “Dads And Daughters Exercising and Empowered” (DADEE) efficacy trial in 2015 [20]. To the authors’ knowledge, this was the first and only physical activity intervention to specifically target fathers and their daughters and only the second to target fathers [19, 20]. The DADEE efficacy trial examined the impact of a physical activity program specifically designed for fathers and their primary school-aged daughters. The program also targeted daughters’ FMS competence, fathers’ and daughters’ screen-time, and fathers’ physical activity parenting practices [20]. Relative to control participants, we observed meaningful and sustained improvements in fathers’ and daughters’ physical activity levels, daughters’ FMS proficiency, fathers’ and daughters’ screen-time and co-physical activity [20].

While these efficacy outcomes are valuable, they were achieved under tightly controlled experimental conditions in a university setting with the program being delivered by the research team. There is a recognized need for efficacy studies to be tested more broadly in generalizable effectiveness trials delivered in a real-world setting [21]. The greater diversity of the participant population, program facilitators, and settings of an effectiveness trial might lead to a decrease in effect of the intervention, a phenomenon known as “voltage drop” [22, 23]. Evaluating the risk of generalizability bias, whereby features of the intervention and sample in the efficacy study are not scalable or generalizable in a larger effectiveness trial, is important in determining the need for program adaptations [24]. Striking a balance between adaptation and maintenance of fidelity of programs may then provide the best opportunities for scale-up and dissemination of evidence-based interventions, thereby maximizing the benefit to overall public health [25, 26].

Therefore, the aim of the current study was to implement and evaluate the DADEE intervention when delivered in community settings by local trained facilitators on the physical activity levels of fathers and daughters and a host of secondary outcomes.

## Methods

### Study Design

We conducted a parallel, two-arm randomized controlled trial with assessments at baseline and post-intervention (3 months post-baseline). In October 2016, father and daughter/s units were randomized using a 1:1 ratio to either (i) the DADEE intervention or (ii) a wait-list control group. The study received institutional approval from the Human Research Ethics Committee and was prospectively registered with the Australian and New Zealand Clinical Trials Registry (ACTRN12616001270404). Prior to program enrollment, fathers provided written, informed consent for themselves and their child. We also obtained child assent.

### Participants

We recruited participants to the free program from across the Newcastle, Australia local government area during September and October 2016 via university and Hunter Medical Research Institute media releases which circulated among several local news outlets (radio, television, newspaper). We also targeted participants via school newsletter advertisements at 69 primary schools located across a diversity of local government areas of socio-economic advantage/disadvantage and social media posts (Facebook, Twitter). Fathers, or father figures (e.g., other male relatives or significant male role-model/friend), aged 18 to 65 years were eligible to participate if they passed a pre-exercise screening questionnaire or provided approval from their general practitioner. Fathers could enroll with one or more daughters. To allow adequate time for completing shared homework tasks, the fathers were required to live with their daughters for at least half of each week. Eligible daughters were aged 4 to 12 years and currently enrolled in primary school (Kindergarten to Year 6).

### The DADEE Intervention

The goal of the DADEE program was to motivate fathers and daughters to become role models and advocates for each other to improve their physical activity. The intervention components and program content were slightly adapted from the original DADEE efficacy program [20] based on process evaluation feedback (e.g., program-length was extended 8-weeks to 9-weeks). The program was developed using evidence from robust qualitative and quantitative studies that targeted fathers [20, 27, 28] and mothers [29] to increase children’s physical activity. To promote participants’ autonomous motivation and enhance the likelihood of sustained behavior change, the program targeted core constructs from self-determination theory (i.e., autonomy, relatedness, competence) and social cognitive theory (e.g., self-efficacy, goal-setting, social support) [30, 31]. Supplementary Table 1 describes intervention components and summarizes the behavior change techniques and associated psychological mediators targeted in each program component.

Participants attended nine consecutive weekly group sessions (90 min) at one of four local primary schools during October to December 2016. The sessions were delivered during after-school hours by the trained facilitators and included three components: (i) a 15-min education session with fathers and daughters, (ii) separate, concurrent 30-min education sessions for fathers and daughters, and (iii) a 45-min practical session for fathers and daughters together. We employed a number of strategies to account for the developmental differences among daughters (aged 4–12 years). Fathers and daughters worked through social-emotional constructs together for the first 15 min each week, allowing fathers to clarify content if required. In addition, program educational content was kept simple, and older girls were paired with younger girls to assist them during education session activities. During the practical component, facilitators described variations of different activities and fathers were trained how to adapt activities so daughters could experience success and remain motivated. To increase family support, we invited mothers and siblings to attend session four, where all family members participated in the activities together.

The father education sessions focused on proven parenting strategies to improve their daughters’ social-emotional well-being, sports skills, and physical activity levels. The daughters’ education sessions targeted the development of key social and emotional skills including self-control, positivity, persistence, critical thinking, resilience, bravery, kindness, and self-reliance. The education sessions provided fathers and daughters with knowledge and skills to identify, navigate and confront the culture of gender prejudice that infiltrates all aspects of girls’ lives, particularly as it relates to their participation in sport and physical activity. The practical sessions included fun co-physical activities focusing on rough and tumble play, aerobic and muscular fitness and FMS (i.e., sport skills). Participants were also provided with resources to assist them to implement and practice what they learned each week at home together. These resources included: a “Father’s Logbook” (containing home activities, e.g., setting SMART goals, tracking physical activity and co-activity, shared activities to nurture the father–daughter relationship); a “Daughters Booklet” (containing instructions for using the DADEE App and tasks related to development of social-emotional skills and physical activity promotion); the DADEE app (containing a variety of fun physical activities for daughters and fathers to complete and track together weekly) and a “Sport Skills Booklet” (containing key teaching points and practice activities relating to the six sports skills). To optimize participant engagement, the program was socio-culturally designed (i.e., created with reference to the behaviors, values, beliefs, and norms common within a population) to specifically appeal to fathers and daughters [31]. For example, to appeal to fathers, the program specifically targeted fathers only [32], was held outside of traditional working hours [33], provided an opportunity for them to spend quality time engaging with their daughters in fun co-physical activities and focused on the benefits of physical activity for girls’ social and emotional well-being [27].

### Program Location and Facilitators

The program was delivered after-hours in local primary schools located in low to middle range socio-economic status areas, on four nights per week. Program facilitators were recruited from attendees of a 3-day facilitator training workshop (15 hr) held at the University of Newcastle in 2016. The workshop was the major component of a teacher education course for pre-service health and physical education or primary teachers. It was also open to local in-service teachers, recruited via school newsletters and emails, and other adults working in health and physical activity related professions, recruited via the Daughters and Dads website. Inservice teachers could earn accredited professional development hours by attending training. During the workshop, participants received instruction on effective delivery of theory and practical sessions to fathers and daughters, and information on the program content. Each program was delivered by trained adult facilitators. To increase relatability, each program included, at a minimum, one male facilitator, who led the father-focused education components, and two female facilitators who led the daughter-focused education components [31]. All facilitators contributed to the components which included both fathers and daughters (e.g., practical sessions).

### Measures

Assessments were completed at baseline (1–4 weeks prior to program commencement) and post-intervention (3 months post-baseline; 1–2 weeks after the program had completed). All data were collected between October and December 2016. The primary outcomes were fathers’ and daughters’ physical activity levels (average steps/day). We chose pedometers to measure the primary outcome because they have good construct validity for measuring physical activity [34], show strong concordance with other physical activity measures [35] and are less expensive per unit relative to accelerometers. We provided participants with Yamax SW200 pedometers (Yamax Corporation, Kumamoto City, Japan) which have been validated in children [36] and adults [37]. Participants were given explicit verbal and written instructions about wearing pedometers, including the expected wear time. They were asked to wear the pedometer for seven consecutive days during all waking hours (except in circumstances where it could get wet or damaged) and to record their steps on a log sheet each day. Mean step counts were only generated for participants with at least four days of pedometry, including one weekend day. Participants were also asked to record any additional physical activity, including the intensity and duration, undertaken when not wearing the pedometer (e.g., swimming). A standardized formula, based on guidelines for children [38, 39] (e.g., 10 min of moderate-to-vigorous-intensity physical activity = 1,200 steps), was used to convert these additional activities into steps. These additional steps were added to the pedometer step count for an adjusted secondary analysis.

A number of secondary outcomes were also assessed and are described in Table 1. Adherence to the program was assessed using rates of session attendance, logbook activity completion, and app usage. Demographic information included participant age, fathers’ education level, employment status, marital status, and country of birth. Socioeconomic status was established using the Australian postal area index of relative socioeconomic advantage and disadvantage (SEIFA) [40]. SEIFA measures the characteristics of an area rather than of individuals. Areas are divided into five equally sized groups of the population and ordered by disadvantage (i.e., quintile 1 = most disadvantaged; quintile 5 = most advantaged) [40].

**Table 1.** Secondary outcomes measured in the DADEE study^a^

Outcome	Description
Fathers only	
Moderate-to-vigorous physical activity (MVPA)	• Average weekly MVPA was measured with a modified version of the *Godin Leisure Time Exercise Questionnaire* [41]. • Overall weekly MVPA was calculated by multiplying self-reported average weekly number of MVPA bouts by average bout length [42].
Physical activity parenting practices	• A number of validated scales including physical activity modeling [43] and co-physical activity (days per week where father and daughters were physically active together) [44] were used to assess parenting practices. • The *Parenting Strategies for Eating and Activity Scale* was used to assess fathers’ control, limit setting, discipline, and monitoring relating to their daughter’s physical activity and screen-time [45].
Co-physical activity	• Co-physical activity was assessed using a scale we developed for this study. It was based on an item from the validated Youth Media Campaign Longitudinal Survey [44] which has been used in previous research [20, 46]. In the current scale, fathers reported how often (number of days/week) and for how long (average minutes/day), they engaged in co-physical activity, with their daughter in the previous week. Separate scales were completed for one-on-one co-activity, and co-activity when other family members were present.
Daughters only	
Fundamental movement skills (FMS)^b^	• The validated *Test of Gross Motor Development* was used to assess FMS competency. Daughters watched a live demonstration of six object control skills (kicking, catching, striking a stationary ball, stationary dribble, overhand throw [TGMD-2], and underhand throw [TGMD-3]) [47] and were then filmed performing each skill twice. • Each skill received a score of 1 or 0 for the presence or absence of certain performance criteria (e.g., bat contacts ball). • The scores for both attempts across all skills were summed to provide the overall object control score.
Perceived competence^c^	• The sports competence scale of the *Physical Self-Description Questionnaire* [48] was used to assess daughters’ perceived sporting competence.
Fathers and daughters	
Screen-time^d^	• Screen-time was measured using a modified version of the *Adolescent Sedentary Activity Questionnaire* [49]. Fathers completed the questionnaire, reporting the total time they and their daughter spent sitting using screens (of any kind) for anything outside of work (or homework when reporting for daughters) on each day in the previous week. • This adapted measure has been used previously in adolescent behavior change research and has shown good sensitivity to change [50].
Weight status	• Weight was measured in light clothing, without shoes on a digital scale to 0.01 kg (model CH-150kp, A&D Mercury Pty Ltd, Australia). • Height was measured using the stretch stature method on an electronic stadiometer to 0.1 cm (model BSM370, Biospace, USA). • For fathers, BMI was calculated dividing weight in kilograms by height in metres squared. • For daughters, BMI-z scores were calculated by using the LMS method (World Health Organization growth reference centiles) [51].
Process measures	• Process outcomes included study retention, average attendance rates, and program satisfaction.

*Notes*

^a^Data collected using online surveys for all secondary outcomes except FMS and weight status (collected objectively at the University of Newcastle by research team).

^b^FMS assessments were undertaken by trained research staff.

^c^Daughters’ questions were interviewer administered one-on-one to ensure comprehension.

^d^Daughter screen-time reported by fathers in relation to eldest enrolled daughter.

### Sample Size

Alpha was set at .025 to adjust for the two primary outcomes. A sample size of 68 fathers was required to provide 80% power to detect a 1,200 step/day difference in physical activity between the intervention and control groups at 3 months with 20% attrition. This calculation was based on a pre-post correlation of .76 and a change score standard deviation of 2,020 steps/day. A sample size of 174 daughters was required to provide 80% power to detect a 1,200 step/day difference in physical activity between the intervention and control groups at 3 months with 20% attrition. This calculation was based on a pre-post correlation of .61 and a change score standard deviation of 2,298 steps/day. Pre-post correlation and standard deviation values were derived from the DADEE efficacy trial data [20].

As multiple daughters per family were eligible to participate, this calculation was also adjusted for clustering at the family level using a correction factor of [1 + (*m*−1) × ICC], where *m* = average daughters per family and ICC = the intra-cluster correlation coefficient. Assuming an average number of 1.33 daughters per family and an ICC of .73 for physical activity, the correction factor is 1.24. Estimates for fathers’ and daughters’ steps and the correction factor for multiple daughters were based on DADEE efficacy trial data [20].

### Randomization

The randomization allocation sequence was generated by a statistician who did not have any contact with participants using a computer-based random number producing algorithm. To ensure evenly matched groups by weight status, since there is an inverse association between BMI and physical activity (a core program element) [52], allocation was stratified by father’s body mass index (BMI) category (18.0–24.9 kg/m^2^; 25.0–29.9 kg/m^2^; 30.0–34.9 kg/m^2^; 35.0–39.9 kg/m^2^; ≥40 kg/m^2^). Group allocation (DADEE intervention or wait-list control) was pre-packed into identical, sealed, opaque, sequentially numbered envelopes according to the randomization schedule by a research assistant who was not involved in enrollment or assessment of participants. After completing baseline assessments, families were allocated to the next available position on the appropriate randomization schedule. Once randomized, intervention group participants were allocated to one of the four local schools for program delivery based on their preference.

### Statistical Analyses

The effectiveness of the DADEE intervention, relative to the control group, was examined using linear mixed models in April 2020 using SPSS 25 (IBM Corp., Armonk, NY). Linear mixed models are robust to the biases of missing data and analysis includes all randomized participants in line with the intention-to-treat principle [53]. The models assessed all outcomes for the impact of group (intervention vs. control), time (categorical), and the group-by-time interaction. To account for clustering of multiple daughters within some families, we used a random intercept for family in analyses of daughters’ self-reported outcomes. Father-reported daughter outcomes only pertained to the eldest daughter; hence this term was not required for models using the fathers’ data.

Prior to the main analyses, we undertook sensitivity analyses using step values truncated to reduce extreme step counts with outcomes demonstrating no difference in significance levels or effect sizes. We conducted additional sensitivity analyses to determine if there were any significant differences in outcomes between participants based on their exposure to the four different weekly program locations and facilitators. As no significant differences were found for any outcomes, this variable was not modeled in the final analyses.

Where significant, we adjusted the analyses for pre-specified covariates, age and socioeconomic status, and the interactions of these covariates with time and group. Effect size was assessed using Cohen’s *d* (mean difference in change divided by the standard deviation of change) [54]. Alpha was set at .025 for analyses of primary outcomes and .05 for all other analyses.

## Results

**Fig 1.**
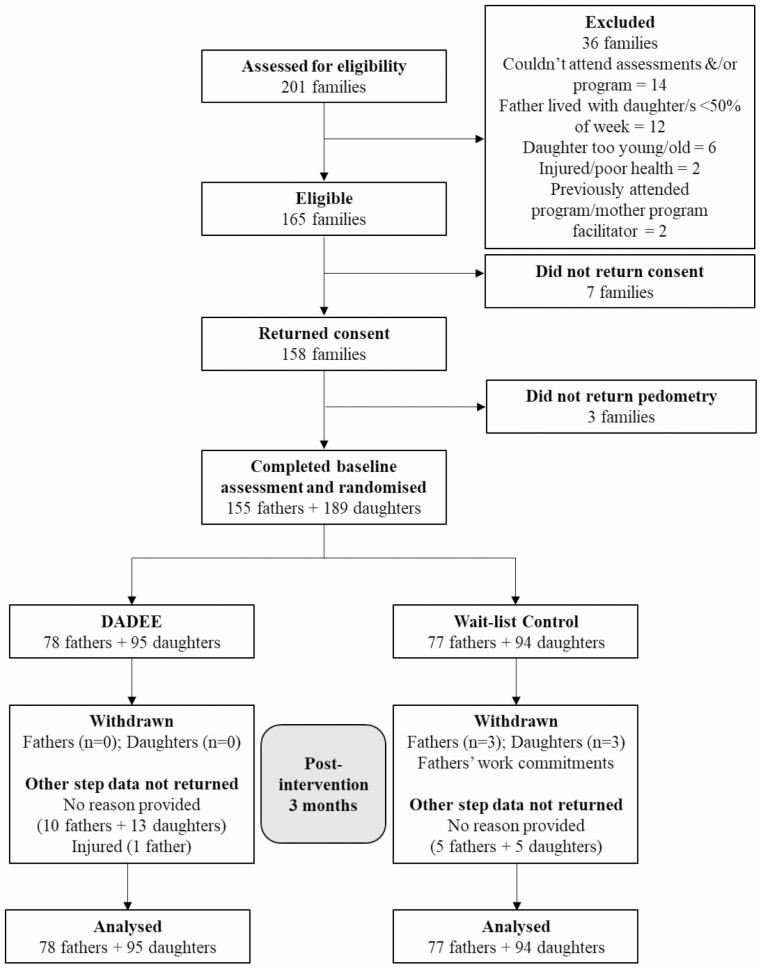
CONSORT diagram describing participant flow through the DADEE community trial for primary outcome.

Baseline assessments were completed by 158 fathers and 193 daughters (*n* = 351) prior to randomization by family unit into the DADEE intervention (78 fathers, 95 daughters) or wait-list control (80 fathers, 98 daughters) groups. Baseline primary outcome data (pedometry) were received from 155 fathers and 189 daughters (*n* = 344) (three fathers and four daughters reported improbable values and therefore not included in analyses) (Fig. 1).

Primary outcome data were collected from 88% of fathers and 89% of daughters at the 3-month primary endpoint. A significantly greater proportion of fathers (*p* = .04,), but not daughters, from the DADEE group (22%) did not return valid pedometer record sheets relative to the control group (10%) at follow-up. There were no significant differences in baseline characteristics between those who did and did not return valid pedometer record sheets at follow-up.

Characteristics of participants at baseline are shown in Table 2. Fathers’ and daughters’ mean ages at baseline were 42.0 (*SD* 5.3) and 8.3 (*SD* 1.8) years, respectively. Most fathers were born in Australia (86%), employed (99%), and married or living with a partner (94%). Families were spread across most socio-economic areas. On average, fathers’ and daughters’ baseline daily step counts were 7,500 (*SD* 2,746) and 9,847 (*SD* 2,748), respectively.

**Table 2.** Demographic and anthropometric characteristics of study participants at baseline

Daughters	Control (*n* = 98)		DADEE (*n* = 95)		Overall (*n* = 193)	
	Mean	*SD*	Mean	*SD*	Mean	*SD*
Age (y)	8.2	1.8	8.4	1.9	8.3	1.8
BMI-z^a,b^	0.26	1.0	0.21	1.04	0.24	1.02
Weight status^b^	*N*	%	*N*	%	*N*	%
* *Underweight	9	9	12	13	21	11
Healthy weight	71	73	66	70	137	72
Overweight	13	13	12	13	25	13
Obesity	4	4	4	4	8	4
Fathers	Control (*n* = 80)		DADEE (*n* = 78)		Overall (*n* = 158)	
	Mean	*SD*	Mean	*SD*	Mean	*SD*
Age (y)	41.8	5.4	42.1	5.3	42.0	5.3
BMI (kg/m^2^)	28.1	4.4	27.7	4.3	27.9	4.3
BMI category^c,d^	*N*	%	*N*	%	*N*	%
Healthy weight	19	24	19	24	38	24
Overweight	42	53	42	54	84	53
Obesity	19	24	17	22	36	23
Post-school qualification^c,d^	75	94	71	91	146	92
Socio-economic status^e^						
Quintile 1 (lowest)	0	0	0	0	0	0
Quintile 2	22	28	14	18	36	23
Quintile 3	37	46	26	33	63	40
Quintile 4	12	15	23	29	35	22
Quintile 5 (highest)	9	11	15	19	24	15
Enrolled daughters per family						
One	80	82	78	82	158	82
Two	18	18	15	16	33	17
Three	0	0	2	2	2	1

*Notes*

^a^BMI-z calculated using the LMS method (World Health Organization growth reference centiles) [51].

^b^Height and weight data used to calculate BMI-z and weight status were collected from 191 daughters overall, Control *n* = 97, DADEE *n* = 94.

^c^BMI categories: healthy weight BMI = <25 kg/m^2^; individual with overweight BMI = 25–30 kg/m^2^; individual with obesity BMI = >30 kg/m^2^.

^d^Trade/apprenticeship, certificate/diploma, university degree, or higher university degree.

^e^Socio-economic status by population quintile for SEIFA Index of Relative Socio-economic Advantage and Disadvantage [55] Area-based quintiles are categorized by dividing the areas, ordered by disadvantage, into five equally sized groups. SEIFA measures the characteristics of an area rather than of individuals. Quintile 1 includes the 20% most disadvantaged areas. Quintile 5 includes the 20% most advantaged areas.

### Primary Outcomes

**Fig 2.**
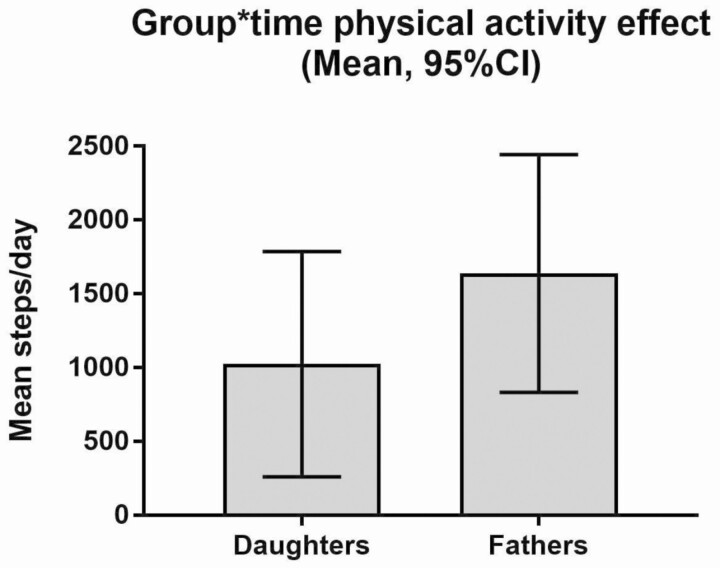
Group by time effects on daughters’ and fathers’ mean daily step count. Data are means and 95% confidence intervals (intention-to-treat).

Relative to the control group, a significant physical activity intervention effect was detected for both fathers (mean difference between groups = +1,638; 95% CI: 833, 2,443) and daughters (mean difference between groups (MD) = +1,023; 95% CI: 259, 1,787) at post-intervention, representing large and medium effect sizes, respectively (Table 3; Fig. 2). Outcomes for adjusted step counts (step counts increased to include equivalent steps for documented non-ambulatory activity) were consistent with those of unadjusted steps for daughters and fathers.

**Table 3.** Changes in primary and secondary outcomes

		Baseline	3-month change from baseline (Mean, 95% CI)		
Outcome	Group	Mean (SE)	Within group^a^	Mean difference between groups^b^	*p*-value [Cohen’s *d*]
Primary outcomes					
Steps/day^c^					
* *Daughters (*n* = 189)	Intervention	9,762 (295)	**+960 (410, 1511)**		
	Control	9,966 (296)	**-63 (-593, 467)**	**1,023 (259, 1,787)**	**.009 [0.4]**
* *Fathers (*n* = 155)	Intervention	7,446 (313)	**+1,962 (1384, 2541)**		
	Control	7,562 (316)	+324 (−235, 884)	**1,638 (833, 2,443)**	**<.001 [0.7]**
Secondary outcomes					
Adjusted steps/day^c,d^					
* *Daughters (*n* = 189)	Intervention	10,808 (362)	**+2,091 (1,310, 2,873)**		
	Control	11,042 (364)	+458 (−298, 1214)	**1,634 (546, 2,721)**	**.003 [0.4]**
* *Fathers (*n* = 155)^e^	Intervention	8,052 (366)	**+1,904 (1,197, 2,611)**		
	Control	8,910 (369)	+280 (−403, 964)	**1,624 (641, 2,607)**	**.001 [0.5]**
Daughters PA (days/week) (*n* = 158)	Intervention	2.3 (0.2)	**+1.0 (0.6, 1.4)**		
	Control	2.5 (0.2)	+0.3 (0.0, 0.7)	**0.7 (0.4, 1.2)**	**.013 [0.4]**
Fathers’ MVPA^g^ (minutes/week) (*n* = 158)	Intervention	143 (14)	**+36 (11, 61)**		
	Control	143 (14)	**+39 (15, 63)**	−3 (−38, 32)	.86 [0.0]
Daughters’ sport competence					
* *Object control score	Intervention	18.4 (0.6)	**+10.4 (9.3, 11.6)**		
* *(TGMD) (*n* = 191)^f^	Control	18.4 (0.6)	**+3.4 (2.4, 4.5)**	**7.0 (5.5, 8.5)**	**<.001 [1.3]**
Perceived sports competence (*n* = 192)^f^	Intervention	4.6 (0.1)	0.01 (−0.12, 0.16)		
	Control	4.6 (0.1)	−0.08 (−0.22, 0.07)	0.1 (−0.1, 0.3)	.42 [0.1]
Screen-time (week day)					
* *Daughters (minutes/day) (*n* = 158)	Intervention	93 (6)	−**28 (**−**38,** −**19)**		
	Control	94 (6)	−8 (−17, 1)	−**20 (**−**34,** −**7)**	**.002 [0.5]**
* *Fathers (minutes/day) (*n* = 158)	Intervention	103 (6)	−**24 (**−**35,** −**13)**		
	Control	114 (6)	−**12 (**−**23,** −**1)**	−12 (−28, 4)	.15 [0.2]
Screen-time (weekend)					
* *Daughters (minutes/day) (*n* = 158)^f^	Intervention	169 (8)	−**39 (**−**53,** −**26)**		
	Control	166 (8)	−2 (−15, 12)	−**38 (**−**57,** −**19)**	**<.001 [0.6]**
* *Fathers (minutes/day) (*n* = 158)	Intervention	146 (129, 162)	−**29 (**−**44,** −**15)**		
	Control	152 (136, 168)	−10 (−25, 4)	−19 (−39, 1.5)	.07 [0.3]
Physical activity parenting practices					
* *Co-PA 1-on-1^f^	Intervention	0.9 (0.1)	**+1.4 (1.0, 1.7)**		
* *(days/week) (*n* = 158)	Control	0.7 (0.1)	**+0.4 (0.1, 0.7)**	**1.0 (0.5, 1.4)**	**<.001 [0.7]**
* *Co-PA family^f, g^	Intervention	1.2 (0.1)	**+1.0 (0.7, 1.3)**		
* *(days/week) (*n* = 158)	Control	1.0 (0.1)	**+0.4 (0.1, 1.0)**	**0.6 (0.2, 1.1)**	**.006 [0.4]**
* *Co-PA 1-on-1^f^	Intervention	34 (7)	**+48 (35, 61)**		
* *(total minutes/week) (*n* = 158)	Control	34 (7)	+12 (−1, 24)	**37 (18, 55)**	**<.001 [0.6]**
* *Co-PA family	Intervention	52 (7)	**+29 (13, 44)**		
* *(total minutes/week) (*n* = 158)	Control	45 (7)	+4 (−11, 20)	**24 (2, 46)**	**.03 [0.3]**
* *Modeling (*n* = 158)^e^	Intervention	2.5 (0.1)	**+0.5 (0.4, 0.6)**		
	Control	2.5 (0.1)	+0.1 (0, 0.2)	**0.4 (0.2, 0.6)**	**<.001 [0.7]**
* *Limit setting (*n* = 158)	Intervention	3.6 (0.1)	**+0.4 (0.2, 0.5)**		
	Control	3.6 (0.1)	+0.2 (0, 0.3)	0.2 (−0.1, 0.4)	.12 [0.3]
* *Monitoring (*n* = 158)	Intervention	2.8 (0.1)	**+0.8 (0.7, 1.0)**		
	Control	2.8 (0.1)	**+0.2 (0.0, 0.3)**	**0.6 (0.4, 0.9)**	**<.001 [0.9]**
* *Disciplining (*n* = 155)	Intervention	2.1 (0.1)	+0.1 (−0.2, 0.3)		
	Control	2.3 (0.1)	−0.1 (−0.2, 0.3)	0.1 (-0.2, 0.5)	.43 [0.1]
* *Control (*n* = 158)	Intervention	2.6 (0.1)	−**0.4 (**−**0.7,** −**0.1)**		
	Control	2.6 (0.1)	−0.1 (−0.3, 0.2)	−0.3 (−0.7, 0.1)	.1 [0.3]
Weight status					
* *Daughters (BMI-z) (*n* = 193)	Intervention	0.2 (0.1)	−0.05 (−0.1, 0.0)		
	Control	0.3 (0.1)	−0.05 (−0.1, 0.0)	0.0 (−0.1, 0.1)	.98 [0.0]
* *Fathers (BMI) (*n* = 158)	Intervention	27.7 (0.5)	−0.2 (−0.7, 0.3)		
	Control	28.1 (0.5)	+0.1 (−0.4, 0.6)	−0.3 (−1.0, 0.4)	.34 [0.2]

*Notes*: Bold denotes a significant difference.

*MVPA* moderate-to-vigorous physical activity; *TGMD* Test of Gross Motor Development; *FMS* fundamental movement skills; *BMI* body mass index.

^a^10-week value minus baseline.

^b^Within-group difference (intervention) minus within-group difference (control).

^c^Criteria for step logs = minimum of 4 days per week including at least one weekend day.

^d^Adjusted to include additional activity completed without wearing pedometer (e.g., swimming).

^e^Adjusted for SES.

^f^Adjusted for daughter’s age.

^g^Adjusted for father’s age.

### Secondary Outcomes

Intervention daughters demonstrated a large and significant improvement in FMS proficiency relative to control daughters (MD = +7.0; 95% CI: 5.5, 8.5). However, there was no concurrent improvement detected in daughters’ perceived sports competence. Daughters also demonstrated significant improvements in several father-reported outcomes including the number of days/week they met physical activity guidelines (MD = +0.7; 95% CI: 0.4, 1.2) and their weekday (MD = −20; 95% CI: −34, −7) and weekend screen-time (MD = −38; 95% CI: −57, −19). Intervention fathers reported significantly better outcomes, relative to control fathers, for a number of parenting practices including the frequency and duration of co-physical activity (both one-on-one [MD = +1.0; 95% CI: 0.5, 1.4] and with other family members [MD = +0.6; 95% CI: 0.2, 1.1]). Improvements in minutes of co-physical activity produced similar results. Intervention effects were also seen for physical activity and screen-time modeling (MD = +0.4; 95% CI: 0.2, 0.6) and monitoring (MD = +0.6; 95% CI: 0.4, 0.9). No significant differences were found for fathers’ self-reported moderate-to-vigorous-intensity physical activity, screen-time, and several parenting practices (i.e., limit-setting, disciplining, control). No differences were detected in fathers’ BMI or daughters’ BMI-z.

### Process Evaluation

A total of 84% of fathers and 82% of daughters attended at least seven out of the nine program sessions. Overall satisfaction with the program and facilitators were assessed by fathers on a scale of 1 (*poor*) to 5 (*excellent*) and both received scores of 4.8 (*SD* 0.4). Independent observers attended program sessions and rated program fidelity (i.e., whether activities were delivered as planned) on at least two occasions at each program location. Program fidelity was high as evidenced by almost all content being delivered (i.e., session slides and practical activities) and observers subjectively rating daughter and dad engagement/enjoyment of the program as 4.7 (*SD* 0.8) and 4.3 (*SD* 0.7) out of 5.0, respectively.

### App Usage and Logbooks

The app was used at least once by 65 (83%) intervention group families. Fathers and daughters completed a median of 13 (IQR: 3, 24; range: 0−53) app activities across the intervention period. Logbooks were returned for evaluation by 66 (85%) intervention group fathers. Physical activity goals for fathers (individually) and fathers and daughters (together) were set by 85% and 78%, respectively. Weekly physical activity levels were recorded on at least seven of nine weeks by 92% of families. On average, 90% of fathers participated in co-physical activity at least once per week and completed two of three weekly daughters and dads tasks each week, despite only being required to complete one.

## Discussion

The primary aim of this study was to evaluate the effectiveness of the DADEE intervention, targeting fathers and their primary-school aged daughters, when delivered in community settings by local trained facilitators. Relative to the control group, both daughters and fathers increased their physical activity levels over 3 months by more than 1,000 and 1,600 steps/day, respectively. The intervention also improved daughters’ FMS proficiency and screen-time, father–daughter co-physical activity, and some fathers’ physical activity-related parenting practices. These findings are comparable with those from our efficacy study when the program was delivered by members of the research team [20].

At baseline, intervention daughters’ daily step counts were below the recommendations for primary-school aged girls (i.e., 10,000 to 11,700 steps/day) [38]. However, by 3 months the intervention group had increased their daily step count by almost 10%, nearly meeting recommendations, whereas the control group had reduced their step count. The physical activity effect size was small-to-moderate and comparable to the efficacy study. Whilst this increase in activity appears modest, it is important to note that most physical activity interventions for children generate only small improvements in objectively measured physical activity (non-significant mean difference in MVPA for intervention group versus control: 1.47 (95% CI −1.88, 4.82) minutes/day) [13, 56, 57]. The increase in activity among daughters in this study is also meaningful when considered in the context of declining levels of MVPA among children which increases with age and is greater for girls than boys [58]. Furthermore, the decline is most pronounced at age 9 (−7.8% MVPA/year for boys vs. −10.2% MVPA/year for girls) which is close to the mean age of the sample in this study [58]. Additionally, even slight improvements in physical activity are associated with health benefits in high-risk children and may have an important impact at the population level [59]. This underscores the importance of this program in addressing the lack of activity interventions targeting girls [60] and calls for interventions to promote MVPA prior to adolescence [58].

The physical activity outcomes for fathers in the current study were also positive, with effect sizes comparable to the DADEE efficacy study [20] and the Healthy Dads Healthy Kids studies [27, 28], which are the only family-based physical activity trials targeting fathers [19]. Compared with the control group, fathers significantly increased their daily step count by more than 1,600 steps.

Our findings for secondary outcomes are intended to complement the primary outcomes and to provide useful insights for future hypothesis testing. Fathers increased their time spent in co-physical activity with their daughters. However, whilst they increased their MVPA by 36 min/week from below recommended levels at baseline to above at 3 months, the group-by-time effect was not significant as the control group also reported a similar increase (39 min/week). This unexpected outcome might be a function of the nature of self-reported MVPA, which is prone to social-desirability bias [61]. Alternatively, intervention fathers may have become more active but spent more time in light physical activity rather than MVPA. Although not significant, intervention fathers reported reductions in screen time on weekdays (−24 min/day) and weekends (−29 min/day). This indicates the presence of role-modeling behavior for their daughters and the time displaced from sedentary behavior may have been utilized for co-physical activities.

Intervention daughters substantially improved their object control FMS proficiency. The large effect size associated with improvements in FMS proficiency in the current study is similar to that found in the DADEE efficacy study [20], which was one of the largest FMS intervention effects reported in the literature [62]. This is important given FMS proficiency is very low among Australian children, and girls have lower FMS proficiency than boys, particularly for object control skills [63]. Childhood FMS proficiency is also positively associated with cardiorespiratory and musculoskeletal fitness and healthy weight status across childhood and adolescence [10, 64]. As such, our positive findings may have a key impact on the girls’ overall health and engagement in physical activity and sport through adolescence and beyond [65, 66].

Despite improvements in actual FMS proficiency, daughters did not improve their perceived sports competence. This finding differs from positive effects of the efficacy study where perceived sports competence improved. While this discrepancy is difficult to explain, it may have been due to the difference between community program facilitators and the University team in their emphasis and explanation of the importance of fathers providing warm and encouraging feedback to their daughters when practicing sports skills and not over-coaching or over-correcting errors. Future facilitator training may need to highlight the importance of imparting this skill to fathers. The impact of the program on daughter’s self-perception may require further investigation/tools to address the discord between improvements in daughters FMS proficiency but no concomitant improvement in their self-reported perception of their sporting abilities.

Both daughters and fathers became more active, and the intervention group daughters significantly reduced their screen time, however there was no change in weight status. These results are consistent with the 3-month outcomes of the DADEE efficacy RCT. It is likely that a longer period of increased activity or targeting dietary intake are required to impact weight status [67] given successful weight loss observed in the Healthy Dads Healthy Kids programs which targets both physical activity *and* diet and targeted weight loss in fathers with overweight and obesity [27, 68].

Program acceptability was demonstrated by high program attendance, retention and program and facilitator satisfaction. Systematic reviews of physical activity interventions for girls and youth have indicated there is a dose–response relationship between attendance rates and physical activity outcomes with a minimum of 40% to 75% attendance being required for improved outcomes [69, 70]. Overall, 84% of fathers and 82% of daughters attended at least seven out of the nine sessions. Although slightly lower than the attendance rate of the efficacy study (93% of fathers and 89% of daughters), this still represents very strong engagement. This is particularly important considering that fathers rarely participate in family interventions and hence suggests that fathers are capable of committing adequately to participation in family interventions [19]. This may be due to careful designing of program content to target and incorporate the unique values and preferences of the target sample (i.e., fathers and daughters) [31] but also suggests that the program delivered in the community by local trained facilitators was highly engaging and delivered with fidelity. Engagement with logbook tasks was generally good with logbook components having an average completion or participation rate of 80% to 92%. However, it should be noted that logbook engagement is unknown for the 15% of intervention group fathers who did not return their logbooks. In contrast, participants used the app infrequently and therefore it may not be a useful inclusion in future DADEE programs.

When moving from the efficacy to the effectiveness stage of an intervention, many programs are affected by “program drift” [23]. This concept involves deviation from the original intended protocols to accommodate a different context and often leads to a “voltage drop” in intervention outcomes [23, 24]. The outcomes in the current study were highly comparable to the efficacy study, which may indicate the high level of fidelity achieved. Further scale-up and dissemination of this program may require some adaptations to best suit other contexts (e.g., developing a train-the-trainer package for government, sport sector or corporate bodies) and diverse populations (e.g., indigenous, disadvantaged) [71]. However, the outcomes of this effectiveness study, which align so closely with the efficacy study, indicate the program in its current form is well-poised for future scale-up and dissemination.

### Strengths and Limitations

This study has a number of strengths such as the capacity to compare outcomes with the efficacy study which will help to inform the development of the program for future large-scale dissemination. Other strengths include incorporation of key recommendations derived from a systematic review of family-based interventions to increase physical activity in children (e.g., goal-setting, co-physical activity) [60] and the novel targeting of daughters and fathers. The capacity to deliver the program effectively using trained facilitators and existing school facilities, augurs well for program scale-up in the future. Additional strengths include the randomized controlled study design, statistical adjustment for clustering of daughters within families, device-based measurement of the physical activity primary outcomes, recruitment of 65% of families of low-to-middle socio-economic status and robust attendance and retention rates. The study also had some limitations. The follow-up timepoint of 3 months post-baseline was relatively short and therefore longer-term effects could not be assessed. Although pedometers capture overall activity, they do not allow assessment of duration or intensity of activity and future research should consider the use of accelerometers. Additionally, despite being delivered in a community setting with a wide range of socio-economic status reported, the recruited sample of fathers who were mostly married or living with a partner, employed, and educated. Future studies should attempt to recruit families with greater diversity.

## Conclusion

The current effectiveness trial, a novel physical activity intervention targeting fathers and their preadolescent daughters, was successfully delivered in a community setting by trained local facilitators with high program fidelity and acceptability. The outcomes for fathers and daughters were comparable to those of the efficacy study, thus establishing the effectiveness of the DADEE intervention in a real-world context. These findings may help to inform the future large-scale dissemination of this evidence-based physical activity intervention into the wider community to maximize the benefit to public health.

## Supplementary Material

kaab056_suppl_Supplementary_Tables
