# Supplementary material for: Establishing Effectiveness of a Community-based, Physical Activity Program for Fathers and Daughters: A Randomized Controlled Trial
Source: Ann Behav Med. 2021 Jul 7;56(7):698–711. doi: 10.1093/abm/kaab056 (PMC9274990; doi:10.1093/abm/kaab056)
Supplement: kaab056_suppl_Supplementary_Tables [file kaab056_suppl_supplementary_tables.docx]

| **Supplementary Table 1**. DADEE^a^ Intervention Components | | | |
| --- | --- | --- | --- |
| Intervention Component | Description | Behavior change techniques for physical activity^c^ | Targeted theoretical mediators |
| Group sessions | Nine weekly 90-minute sessions conducted including:   - *15-minute combined education session* for fathers and daughters including an overview of the weekly content and shared activities. - *30-minute education session for fathers* delivered by a trained male facilitator covering key concepts summarised in Supplementary table 2. - *30-minute education session for daughters* delivered by two trained female facilitators providing age-appropriate content promoting increasing activity and the development of social-emotional skills. - *45-minute practical session* for fathers and daughters together involving engaging physical activities in a positive and supportive atmosphere with the intent to increase daughters’ intrinsic motivation to be physically active. The activities included rough and tumble play, sports skills (i.e., FMS^b^) and aerobic and muscular fitness which were incorporated into fun, father-daughter games and challenges. | - Social support (emotional, practical) - Increase positive emotions^d^ - Instructions on how to perform a behavior - Information about consequences (health, emotional, social & environmental) - Demonstration of a behavior - Graded tasks - Credible source - Identification of self as a role model - Framing/re-framing - Verbal persuasion about capability | - Social support/relatedness (SCT/SDT) - Autonomy (SDT) - Self-efficacy/perceived competence (SCT/SDT) - Outcome expectations (SCT) - Goals (SCT) |
| Father resources | - *Father’s Logbook* which included tasks for completion at home involving setting SMART goals, tracking fathers’ and daughters’ physical activity and co-activity, and nurturing the father-daughter relationship. - *Dads’ Session Slides Booklet* containing copies of the content presented at the weekly fathers’ education sessions. | - Goal setting - Action planning - Self-monitoring - Self-incentive - Non-specific incentive - Social support (practical, emotional) | - Goals (SCT) - Social support/relatedness (SCT/SDT) |
| Daughter Resources | - *Daughters Booklet* which included instructions for using the DADEE App and weekly tasks for completion at home. *Word of the Week tasks* related to improving their understanding of social-emotional skills (e.g., self-control, persistence, positivity, resilience, critical thinking, kindness) were completed to earn stickers. - *Em Power Collector Cards* were awarded weekly to daughters by their fathers after they completed a variety of fun physical activities together. | - Instructions on how to perform a behavior - Graded tasks - Self-talk - Material incentive (behavior) - Increase positive emotions^d^ | - Social support/relatedness (SCT/SDT) - Goals (SCT) - Autonomy (SDT) - Self-efficacy/perceived competence (SCT/SDT) |
| Family resources | - *Pedometers* (1 x Yamax SX200 per participant) to assist with monitoring physical activity. - *DADEE web-based App* which included instructions for all the Em Power home task activities. - *Sports Pathway Booklet* to help locate and access a wide range of sporting opportunities in the local area. - *Sport Skills Booklet* providing key teaching points and photographic examples for performing six sport skills. - *Program accessories* including T-shirts, water bottles and backpacks. | - Adding objects to the environment - Self-monitoring - Increase positive emotions^b^ - Graded tasks - Self-talk - Prompt/cues - Instructions on how to perform a behavior | - Social support/relatedness (SCT/SDT) - Self-efficacy/perceived competence (SCT/SDT) - Autonomy (SDT) - Goals (SCT) |

Notes: a. DADEE: “Dads And Daughters Exercising and Empowered”; b. FMS: fundamental movement skills; c. Techniques outlined in the “behavior change technique taxonomy” (v1) [1]; d. Technique to be included in future revision of behavior change technique taxonomy [1].

| **Supplementary Table 2** Summary of session content in the DADEE program.^a,b^ | |
| --- | --- |
| Session theme | Key topics |
| 1. Rough and tumble play | - Impact of fathers on girls social-emotional and physical wellbeing - Benefits of rough and tumble play - *Targeted social-emotional construct*: Self-control |
|  |  |
|  |  |
| 2. Fitness and physical activity | - Benefits of physical activity - Promoting physical activity through the day - Importance of co-physical activity - *Targeted social-emotional construct*: Positivity |
| 3. Sports skills | - Importance of fundamental movement skills (FMS) - Creating effective and engaging FMS sessions - *Targeted social-emotional construct*: Persistence |
| 4. Female role models^c^ | - Beauty issues in girls - Identifying gender-prejudice - Pinkification vs. equalist parenting - Positive female role models - *Targeted social-emotional construct*: Critical thinking |
| 5. Challenge and adventure | - Authoritative parenting - Reducing screen-time - *Targeted social-emotional construct*: Resilience |
| 6. Home and family spirit | - How to be an involved father - Role of quality and quantity time - Benefits of family dinners - Encouraging independence through self-reliance - *Targeted social-emotional construct*: Self-reliance |
| 7. Community spirit | - Emotional mirroring - Importance of risk taking - *Targeted social-emotional construct*: Kindness |
| 8. Summary | - Program recap - *Targeted social-emotional construct*: Brave and daring |
| 9. Finale | - Revisiting aims and quiz - Dad and daughter presentations - *Review of all social-emotional constructs* |
| 1. DADEE:” Daughters and Dads Active and Empowered”; b. Adapted from: Young et al., Impact of a Father–Daughter Physical Activity Program on Girls’ Social–Emotional Well-Being: A Randomized Controlled Trial [2]; c. Mothers and siblings were invited to participate in this session. | |

**References**

1. Michie S, Richardson M, Johnston M, et al. The Behavior Change Technique Taxonomy (v1) of 93 Hierarchically Clustered Techniques: Building an International Consensus for the Reporting of Behavior Change Interventions. Ann Behav Med. 2013;46(1):81-95. doi: 10.1007/s12160-013-9486-6.

2. Young MD, Lubans DR, Barnes AT, Eather N, Pollock ER, Morgan PJ. Impact of a father–daughter physical activity program on girls’ social–emotional well-being: A randomized controlled trial. J Consult Clin Psychol. 2019;87(3):294.
